# Smart sustainable bottle (SSB) system for *E. coli* based recombinant protein production

**DOI:** 10.1186/s12934-014-0153-9

**Published:** 2014-11-05

**Authors:** Zhaopeng Li, Bettina Carstensen, Ursula Rinas

**Affiliations:** Leibniz University of Hannover, Technical Chemistry – Life Science, Hannover, Germany; Helmholtz Centre for Infection Research, Inhoffenstraße 7, D-38124 Braunschweig, Germany

**Keywords:** *Escherichia coli*, Self-made bioreactor, Autoinduction, Oxygen transfer, k_L_a

## Abstract

**Background:**

Recombinant proteins are usually required in laboratories interested in the protein but not in the production process itself. Thus, technical equipment which is easy to handle and straight forward protein production procedures are of great benefit to those laboratories. Companies selling single use cultivation bags and bioreactors are trying to satisfy at least part of these needs. However, single-use systems can contribute to major costs which might be acceptable when “good manufacturing practices” are required but not acceptable for most laboratories facing tight funding.

**Results:**

The assembly and application of a simple self-made “smart sustainable bottle” (SSB) system for *E. coli* based protein production is presented. The core of the SSB system is a 2-L glass bottle which is operated at constant temperature, air flow, and stirrer speed without measurement and control of pH and dissolved oxygen. Oxygen transfer capacities are in the range as in conventional bioreactors operated at intermediate aeration rates and by far exceed those found in conventional shaking flasks and disposable bioreactors. The SSB system was applied for the production of various recombinant proteins using T7-based expression systems and a defined autoinduction medium. The production performance regarding amount and solubility of proteins with robust and delicate properties was as good as in state-of-the-art stirred tank commercial bioreactors.

**Conclusions:**

The SSB system represents a low cost protein production device applicable for easy, effective, and reproducible recombinant protein production.

**Electronic supplementary material:**

The online version of this article (doi:10.1186/s12934-014-0153-9) contains supplementary material, which is available to authorized users.

## Background

*E. coli* based recombinant protein production is nowadays a routine procedure and small scale production in shake flask cultures can be easily accomplished in most laboratories. However, if larger protein quantities are required most laboratories usually reach their limits and either have to establish collaborations with laboratories dedicated to protein production or have to establish own production facilities generally requiring sophisticated bioreactors and well-trained dedicated personal to run them. Alternatively, single-use systems are nowadays on the market [1,2] which are easy to operate but costly to purchase.

Here, we describe in detail the set-up and assembly of a “smart sustainable bottle” (SSB) system designed for larger scale recombinant protein production using *E. coli* T7-based expression systems. Moreover, we compare the performance of the SSB system with conventional well-equipped bioreactors regarding oxygen transfer and production of recombinant proteins with robust and delicate properties regarding product yield and solubility.

## Results

### Design of the “smart sustainable bottle” (SSB) system

**Figure 1:**
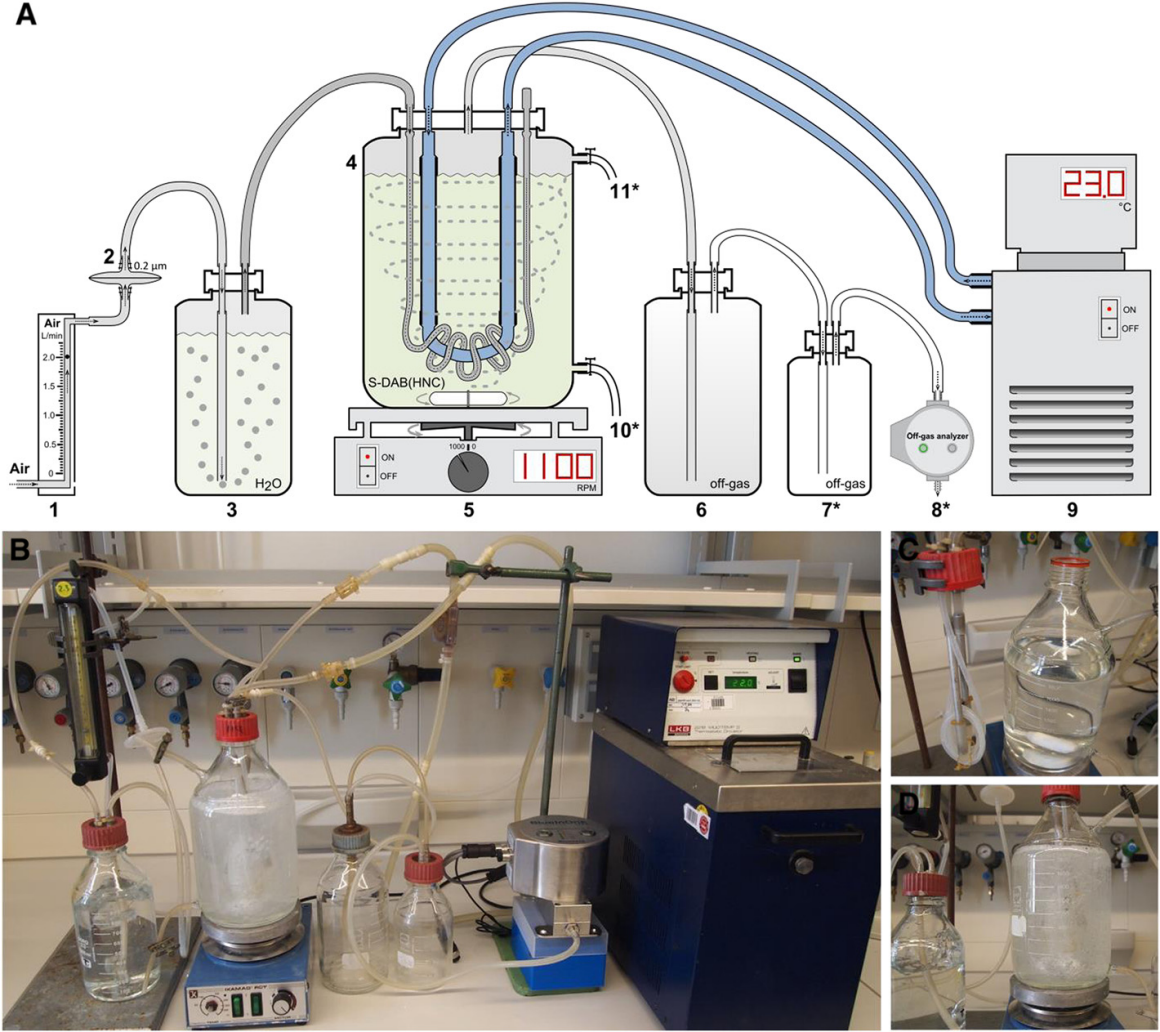
**Schematic diagram and photos of the SSB system.** Schematic diagram of SSB system **(A)**: flowmeter **(1)**, 0.2 μm venting filter **(2)**, 1-L pre-wetting bottle **(3)**, 2-L cultivation bottle **(4)**, magnetic stirrer **(5)**, 1-L safety bottle for off-gas **(6)**, optional second 0.5-L safety bottle for off-gas **(7** ^*^ **)**, optional off-gas analyzer **(8** ^*^ **)**, circulating thermostat **(9)**, optional sample port **(10** ^*^ **)**, optional liquid inlet port **(11** ^*^ **)**. Devices marked with an asterisk are optional and not required for successful operation of the SSB system. Overview of the SSB system **(B)**, sparger and cooling finger **(C)**, and pre-wetting and cultivation bottles **(D)**. For details of the assembly of the SSB system please refer to Additional file 1.

The core of the SSB system is a 2-L glass bottle which is operated at constant temperature, constant air flow, and constant stirrer speed without measurement and control of pH and dissolved oxygen (Figure 1). The temperature is controlled via a self-made cooling finger connected to a conventional circulating thermostat. The airflow is controlled via a conventional flow meter and the inlet air passed through a 0.2 μm venting sterile filter and subsequently moistened by passing through a bottle containing sterile water to prevent water loss from the main bottle during long-term cultivation. The outlet air from the main bottle is passed through a 1-L empty safety bottle and if there is interest to connect an off-gas analyzer to the SSB system it is recommended to add a second safety bottle to protect the analyzer in case excessive foaming occurs. Aeration of the culture broth occurs by passing the air through a self-made sparger and by mixing using a conventional magnetic stirrer. For details of the assembly of the SSB system please refer to Additional file 1.

### Oxygen transfer in the SSB system

The transfer of sufficient oxygen into the medium to allow aerobic cell metabolism is a critical factor in recombinant *E. coli* cultivations. To characterize the oxygen transfer capacities of the SSB system, the volumetric oxygen transfer coefficient (k_L_a) was determined. For comparison, the k_L_a values were additionally determined in shake flasks with and without baffles as well as in a 2-L conventional bioreactor equipped with two Rushton 6-blade impellers but without baffle cage (Table 1). The data show that the oxygen transfer is considerably better in the SSB system compared to commonly employed shake flasks (working volume 1/10 of the flask volume) and is also better than in the 2-L bioreactor run at an agitation speed of 500 rpm (Table 1). However, the bioreactor run at an agitation speed of 1000 rpm outperforms the SSB system (Table 1). Thus, the SSB system is presumably well suited for intermediate (OD_600_ ~ 25) but not for higher cell density cultivations (OD_600_ > 50).

**Table 1:** **Volumetric oxygen transfer coefficient k**_**L**_ **a in shake flask, bioreactor, and SSB system** ^*^

**Cultivation vessel**	**Oxygenation condition**	**k**_**L**_ **a**
Erlenmeyer flask without baffles	160 rpm in a shaker with amplitude of 5 cm	127 h^−1^
Erlenmeyer flask with three baffles	160 rpm in a shaker with amplitude of 5 cm	146 h^−1^
Bioreactor (2-L)	Air flow rate 2 L min^−1^, 500 rpm with two Rushton 6-blade impellers	162 h^−1^
Bioreactor (2-L)	Air flow rate 2 L min^−1^, 1000 rpm with two Rushton 6-blade impellers	701 h^−1^
SSB system (2-L)	Air flow rate 2 L min^−1^, 1100 rpm using a magnetic stirrer bar (Ø 20 mm, L 50 mm)	208 h^−1^

^*^A more detailed literature survey of oxygen transfer capacities in shake flasks, single use culture vessels, and conventional bioreactors is given in Additional file 3: Table S1.

### Comparative production of GFP in a conventional bioreactor and the SSB system

**Figure 2:**
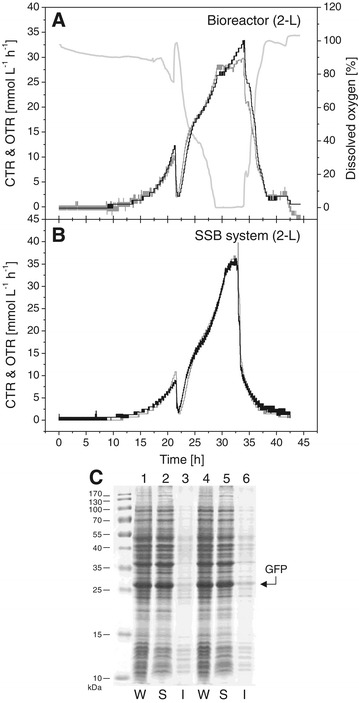
**Production of GFP in a conventional 2-L bioreactor and the SSB system.** Autoinduction cultivations were carried out in a 2-L bioreactor **(A)** and the SSB system **(B)** for the production of GFP. Carbon dioxide (CTR, black line) and oxygen transfer rates (OTR, gray line), and dissolved oxygen concentration (light gray line) are shown. GFP production in the bioreactor (lanes **1**-**3**) and SSB system (lanes **4**-**6**) was analyzed by SDS-PAGE. W: whole cell protein, S: soluble, and I: insoluble part of whole cell protein **(C)**.

The green fluorescent protein (GFP, 27 kDa) is a popular reporter protein with robust production properties concerning solubility and yield [3]. For comparative purposes GFP production was carried out in a conventional 2-L bioreactor equipped with two Rushton 6-blade impellers (run at 500 rpm) and the SSB system (working volume 2-L) using an optimized defined autoinduction medium (Figure 2). This medium results in intermediate cell densities (OD_600_ ~ 20) and omits inducer addition when using T7-based protein production systems [3,4]. Cell metabolism of the recombinant cells as judged by their respiratory activities as well as specific product yields and product solubility were similar in both production vessels (Figure 2). However, during GFP production in the bioreactor oxygen limiting conditions occurred temporarily leading to decelerating metabolic (respiratory) activities (Figure 2A) as compared to the cultivation in the SSB system (Figure 2B) and, thus, slightly lower cell densities and GFP yields (fluorescence) were reached in the bioreactor as compared to the SSB system (Table 2).

**Table 2:** **Recombinant protein production in bioreactor and SSB system**

**Protein**	**Cultivation vessel**	**OD**_**600**_	**Final pH**	**Expression level [%]** ^**1**^		**Fluorescence** ^**2**^	**Fluorescence/OD**_**600**_^**3**^
				**W**	**S**		
GFP	Bioreactor (2-L) _constant agitation (500 rpm)_	17.6 ± 0.5	6.8	8	9	15420 ± 120	857 ± 25
	SSB system (2-L)	20.6 ± 0.3	5.9	8	9	18172 ± 140	865 ± 14
hFGF-2	Bioreactor (2-L) _constant agitation (500 rpm)_	18.8 ± 0.5	6.8	9	8		
	Bioreactor (30-L) _dissolved oxygen-stat (50%)_^#^	18.0 ± 0.6	6.8	8	7		
	SSB system (2-L)	18.9 ± 0.3	5.9	9	7		
	SSB system (2-L)	21.0 ± 0.4	6.0	9	8		
GST-GFP	Bioreactor (2-L) _constant agitation (500 rpm)_	22.3 ± 0.1	6.8	10	7	11569 ± 163	526 ± 8
	SSB system (2-L)	24.7 ± 0.2	5.8	10	6	13044 ± 102	522 ± 6
	SSB system (2-L) with “booster” amino acids	26.2 ± 0.2	6.5	10	9	17981 ± 158	692 ± 8
TRX-hLIF	Bioreactor (2-L) _constant agitation (500 rpm)_	16.0 ± 0.2	6.8	7	1		
	Bioreactor (2-L) _dissolved oxygen-stat (30%)_	18.9 ± 0.2	6.8	8, 7^*^	2, 3^*^		
	SSB system (2-L)	19.0 ± 0.3	5.9	7	1		
	SSB system (2-L)	18.9 ± 0.3	6.0	7	2		
	SSB system (2-L) with “booster” amino acids	23.2 ± 0.4	6.6	7, 7^*^	3, 7^*^		
	SSB system (2-L) with “booster” amino acids	22.6 ± 0.1	6.5	8	3		

^1^Percentage of target protein in whole cell protein (W) and soluble fraction of whole cell protein (S). ^2^Volumetric GFP fluorescence. ^3^Cell specific GFP fluorescence. ^#^Details of comparative hFGF-2 production in a conventional 30-L bioreactor at 50% air saturation and the SSB system are shown in Additional file 2: Figure S1. ^*^Cells were disrupted by high pressure homogenization.

### Comparative production of hFGF-2 in a conventional bioreactor and the SSB system

**Figure 3:**
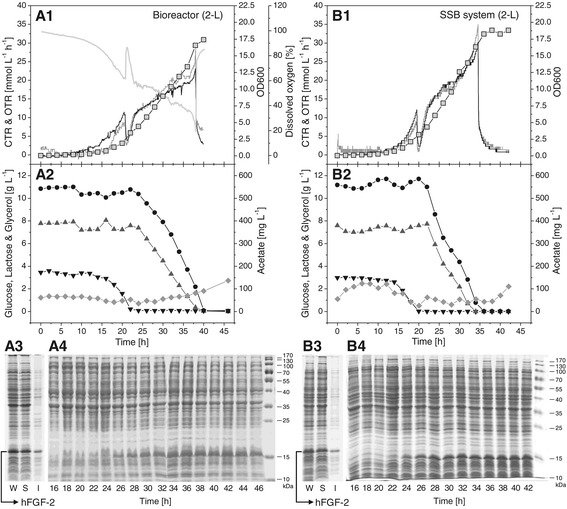
**Production of hFGF-2 in a conventional 2-L bioreactor and the SSB system.** Autoinduction cultivations were carried out in a 2-L bioreactor **(A1**-**A4)** and the SSB system **(B1-B4)** for the production of hFGF-2. Carbon dioxide (CTR, black line) and oxygen transfer rates (OTR, gray line), dissolved oxygen concentration (light gray line), and cell growth (squares) are shown **(A1, B1)**. Moreover, the consumption of glucose (black down triangle), lactose (dark gray up triangle), and glycerol (black circle) as well as the production of acetate (gray diamond) are given **(A2, B2)**. hFGF-2 production in the bioreactor and SSB system was analyzed by SDS-PAGE. W: whole cell protein, S: soluble, and I: insoluble part of whole cell protein **(A3, B3)**. Kinetics of product formation were analyzed by SDS-PAGE of the whole cell protein **(A4, B4)**.

Human basic fibroblast growth factor (hFGF-2, 18 kDa) is a cytokine which can be produced as soluble protein but also in form of inclusion bodies depending on the culture conditions employed [5-7]. The comparative production of hFGF-2 was also carried out in the conventional 2-L bioreactor and the SSB system using the autoinduction medium (Figure 3). Again, respiratory activities of the recombinant cells producing hFGF-2 as well as product yields and product solubility were similar in both production vessels (Figure 3, Table 2). Moreover, comparable kinetics of substrates utilization, acetate formation, cell growth and hFGF-2 production were observed in the bioreactor as well as in the SSB system (Figure 3).

### Generation of difficult-to-produce proteins in the SSB system

**Figure 4:**
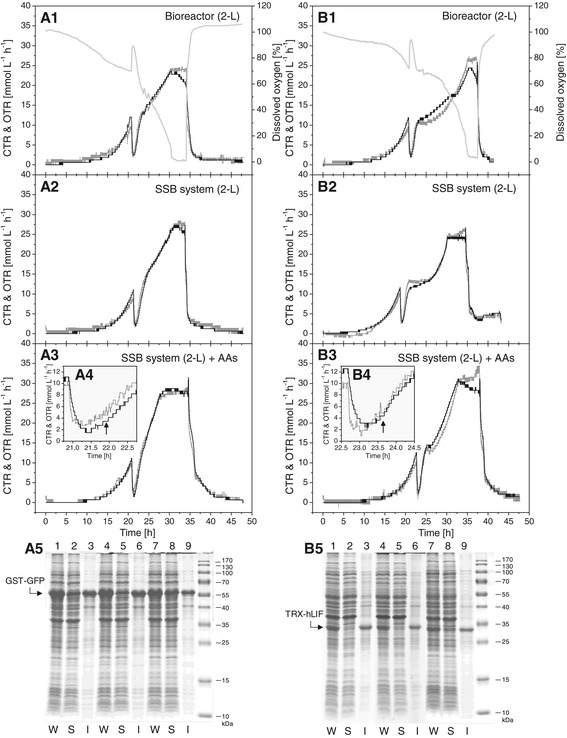
**Production of GST-GFP and TRX-hLIF in a conventional 2-L bioreactor and the SSB system with “booster” amino acids addition.** Autoinduction cultivations for the production of GST-GFP **(A1-A5)** and TRX-hLIF **(B1**-**B5)** were carried out in 2-L bioreactor **(A1 and B1)**, the SSB system **(A2 and B2)**, and the SSB system with “booster” amino acids addition **(A3/4 and B3/4)**. The carbon dioxide (CTR, black line) and oxygen transfer rates (OTR, gray line), and the dissolved oxygen concentration (light gray line) are shown. “Booster” amino acids additions are indicated by arrows in **A4** and **B4**. GST-GFP **(A5)** and TRX-hLIF **(B5)** production in a 2-L bioreactor **(lanes** **1**-**3)**, the SSB system **(lanes 4**-**6)**, and the SSB system with “booster” amino acids addition **(lanes 7**-**9)** were analyzed by SDS-PAGE. W: whole cell protein, S: soluble part, and I: insoluble part of whole cell protein.

Some proteins are difficult to produce as soluble bioactive proteins using *E. coli* as expression system. Examples are the reporter protein GFP carrying an N-terminal glutathione-S-transferase (GST) tag which strongly increases the GFP propensity to form inclusion bodies [3] and the poorly soluble human leukemia inhibitory factor (hLIF) carrying an N-terminal thioredoxin (TRX) tag [8]. The production of both proteins was carried out in the conventional 2-L bioreactor and in the SSB system (Figure 4). Again, the respiratory activities of producing cells were similar in both vessels, however, the production of each protein led to protein-specific respiratory profiles independent of the production vessel (Figure 4). SDS-PAGE analysis revealed very low amounts of both proteins in the soluble cell fraction independent of the production vessel employed (Figure 4).

Previous studies revealed that enzymes utilized for amino acid degradation (e.g. for arginine, threonine or proline) increased in abundance during entry into stationary phase [9], prompting us to investigate the addition of amino acids for improving the soluble production of difficult proteins, namely GST-GFP and TRX-LIF. A mixture of “booster” amino acids (arginine, asparagine, glycine, proline, leucine, and threonine, 10 mM each) was added to the medium. The composition of the amino acid mixture and the timing of addition were determined in preceding experiments (Additional file 2: Figures S2 and S3). Addition of these amino acids after the consumption of glucose and the onset of glycerol/lactose consumption, respectively the recovery of the respiratory activity, improved the soluble production of these proteins considerably (Figure 4, see also for more details Additional file 2: Figures S4 and S5). However, the exact timing is important, appr. 45 min after the recovery of the respiratory activity, as earlier addition did not improve the soluble production of the target protein but rather increased the final cell density and overall product yield (data not shown). After the consumption of all carbon substrates (at about 36 h), the fluorescence of GST-GFP per unit of biomass still increased for about 12 h, indicating the best harvest time is at 48 h (Additional file 2: Figure S4).

## Conclusions

Protein production in the SSB system (2-L) using the defined autoinduction medium (S-DAB (HNC)) leads to equal results concerning final product yields and solubility as reached in conventional bioreactors of identical working volume run at intermediate aeration rates or even as reached in larger bioreactors (30-L) and bioreactors operated at constant dissolved oxygen concentrations (30 or 50% air saturation) (Table 2, Additional file 2: Figure S1). Thus, the SSB system in combination with the autoinduction method represents a very convenient way to carry out recombinant protein production at low cost in batch mode at constant temperature, without oxygen and pH control as well as without inducer addition [3,10]. The oxygen transfer capacity in the SSB system is higher than in conventional baffled shake flasks as well as in the novel single use culture vessels, and in the range as in conventional bioreactors operated at intermediate aeration rates (Table 1, Additional file 3: Table S1). Furthermore, it is possible to increase the solubility of difficult-to-produce proteins in the SSB system (as well as in conventional bioreactors) by the addition of “booster” amino acids. For this purpose, the usage of an off-gas (or dissolved oxygen) analysis system is advised to determine the best time of addition. However, this benefit must be carefully weighed against the additional complexity and cost. In the general, the SSB system can be successfully operated without any measurements (e.g. OD_600_, pH, dissolved oxygen concentration, off-gas, substrates).

## Material and methods

### Strains and media

*E. coli* BL21 (DE3) strains harboring the plasmids pET-28c-His6-GFP [3], pETM30-His6-GST-GFP [3], pET-29c-hFGF-2 [6], and pET32b-trx-his-tev-hLIF [8] were used for the production of GFP, GST-GFP, hFGF-2, and TRX-hLIF, respectively. Cells were grown on the defined autoinduction medium S-DAB (HNC) containing as carbon substrates 2.9 g L^−1^ glucose, 11.2 g L^−1^ glycerol, and 7.4 g L^−1^ lactose. The medium was prepared as described previously [3,4] with slight modifications of the preparation methods (Additional file 4). It can be added to the SSB system in a clean bench or through an optional liquid inlet port (see Figure 1). To prevent foaming 0.25 mL L^−1^ TEGO Antifoam KS 911 (Evonik, Germany) were added to the medium. Appropriate antibiotics were added for plasmid maintenance (pET32b-trx-his-tev-hLIF, 100 mg L^−1^ ampicillin; pET-28c-His6-GFP, pETM30-His6-GST-GFP, and pET-29c-hFGF-2, 50 mg L^−1^ kanamycin). In some experiments, the S-DAB (HNC) medium was supplemented with a “booster” amino acid solution (each amino acid 10 mM final concentration). The “booster” amino acid stock solution containing 125 mM of each L-arginine, L-asparagine, glycine, L-proline, L-leucine, and L-threonine was adjusted to pH 7.5 using HCl prior to sterile filtration. Composition and preparation of Luria-Bertani (LB) and Defined Non-inducing Broth (DNB) for precultures are described in Additional file 4.

### Cultivation conditions

Precultures were prepared as described previously [3]: briefly, a single colony from LB agar plate was transferred to LB medium; after overnight cultivation at 30°C, DNB medium was inoculated with LB medium preculture to give a starting OD_600_ of 0.04; this culture was shaken at 30°C for 6 ∼ 8 h until the OD_600_ reached 1.5 ∼ 2.0 and used to inoculate the main culture using the defined autoinduction medium S-DAB (HNC) with a starting OD_600_ of 0.02 in the SSB system, the 2-L bioreactor (BIOSTAT® Aplus without baffle cage, Sartorius, Germany), and the 30-L bioreactor (Biostat UD, B. Braun Biotech., Germany). Inoculation of the SSB system can be carried out using a clean bench or through the optional liquid inlet port (see Figure 1). The temperature was set to 23°C and the air flow rate to 1 vvm. Temperature control in the SSB system was carried out manually using a circulating thermostat and an infrared thermometer (MiniSight, Optris, Germany) for temperature measurement. The pH was maintained at pH 6.8 in the bioreactor cultivations by automatic addition of NaOH. The pH was not controlled in the SSB system. Due to the high buffering capacity of the S-DAB (HNC) medium, the pH merely dropped from pH 6.8 at the beginning to ~ pH 6 at the end of the cultivation (Table 2). The agitation speeds were 1100 rpm for the SSB system (using a magnetic stirrer bar, Ø 20 mm, length 50 mm) and 500 rpm for 2-L bioreactor (two Rushton 6-blade impellers). In some bioreactor cultivations, the dissolved oxygen concentration was maintained at 30% (2-L bioreactor) and 50% air saturation (30-L bioreactor) through automatic agitation speed control. For the production of difficult-to-produce proteins, “booster” amino acids were added in some cultivations to a final concentration of 10 mM each. The time point of addition was appr. 45 min after the rerise in the carbon dioxide and oxygen transfer rates (respectively, the redecline in the dissolved oxygen concentration) appr. 20 h after inoculation. In this case, the starting volume in the culture vessel should be decreased (i.e. 1.8 L in the SSB system). Cells were harvested after 48 h of cultivation and cell pellets were stored at −80°C until further processing.

### Analytical procedures

Cell growth was monitored by measurement of the absorbance at 600 nm (OD_600_). Off-gas analysis was performed using the BlueInOne Ferm system (BlueSens, Germany). The carbon dioxide and oxygen transfer rates were calculated as described previously [11]. (GST)-GFP fluorescence was measured in triplicate using the F-7000 fluorescence spectrophotometer (Hitachi, Japan) with 395 nm excitation and 510 nm emission filters as described before [12] and according to equipment manufacturer's instructions. For preparation of cell extracts and determination of soluble and insoluble product fractions, cells were disrupted by BugBuster™ Protein Extraction Reagent (Novagen, USA) with rLysozyme and Benzonase according to manufacturer's instructions if not otherwise indicated. Soluble and insoluble cell fractions were separated by centrifugation at 17,000 × g and 4°C for 30 min. SDS-PAGE analysis was performed in the Mini-PROTEAN Tetra Cell (Bio-Rad, USA) according to standard procedures [13] and manufacturer’s instructions. After electrophoresis, proteins were visualized by colloidal Coomassie G-250 staining [14] and the amount of target protein in the total and soluble cell fraction quantified by densitometry using ImageJ software.

For glucose analysis, the YSI 2300 STAT Plus™ glucose & lactate analyzer (YSI Life Sciences, USA) was used. Glycerol and lactose were analyzed using an HPLC system (Agilent technologies, USA). Column temperature (Aminex HPX-87H, BioRad, USA) was set at 65°C and elution carried out with 5 mM H_2_SO_4_ at a flow rate of 0.7 mL min^−1^. Peaks were detected by refractive index detector. Acetate was analyzed by gas chromatography (GC-2010 Plus system, Shimadzu, Japan) using a Nukol™ fused-silica capillary column (Supelco Deutschland GmbH, Germany). The injection temperature was 250°C and the flame ionization detector temperature kept at 280°C. Hydrogen was used as carrier gas at a flow rate of 30 mL min^−1^. During analysis, the column temperature profile was programmed from 100 to 200°C with 10°C steps per minute.

The volumetric oxygen transfer coefficient (k_L_a) was determined by the sodium sulfite oxidation method [15-18] at room temperature. The SSB system, the 2-L bioreactor, and the Erlenmeyer flasks without and with three baffles were filled with a 0.9 M sodium sulfite solution containing 0.6 mM Co^2+^ (CoCl_2_.6H_2_O). The conditions were as follows: air flow rate at 2 L min^−1^ (1 vvm), agitation speeds at 1100 rpm for the SSB system and 500 as well as 1000 rpm for the 2-L bioreactor. The shaking speed for Erlenmeyer flasks were 160 rpm using a shaker with an amplitude of 5 cm (Certomat BS-1, B. Braun Biotech., Germany). Every 15 min, 1 mL samples were taken and mixed with an excess of standard iodine reagent. The amount of residual sulfite was titrated with standard sodium thiosulfate solution to a starch indicator end point and the rate of sulfite consumption was used to determine the k_L_a value as follows [15]:

$$ -\frac{{\mathrm{dC}}_{{\mathrm{Na}}_2{\mathrm{SO}}_3}}{\mathrm{dt}}=2{\mathrm{k}}_{\mathrm{L}}{\mathrm{aC}}^{*} $$

using 0.561 mM as C^*^ for the saturated dissolved oxygen concentration in the liquid phase [19].

## Additional files

Additional file 1
**Details of the assembly of the SSB system.**
Additional file 2: Figure S1 hFGF-2 production in the SSB system and 30-L conventional bioreactor at 50% air saturation. **Figures S2-S3.** Adding “booster” amino acids to improve the soluble production of GST-GFP and TRX-hLIF in shake flask cultivation. **Figures S4-S5.** Adding “booster” amino acids to improve the soluble production of GST-GFP and TRX-hLIF in the SSB system. Additional file 3: Table S1 Literature survey of oxygen transfer capacities (k_L_a values) in shake flasks, single use culture vessels, and conventional bioreactors. Additional file 4
**Details of medium composition and preparation.**
