# Supplementary material for: Smart sustainable bottle (SSB) system for E. coli based recombinant protein production
Source: Microb Cell Fact. 2014 Nov 5;13:153. doi: 10.1186/s12934-014-0153-9 (PMC4226889; doi:10.1186/s12934-014-0153-9)
Supplement: Additional file 1 — Details of the assembly of the SSB system. [file 12934_2014_153_MOESM1_ESM.pdf]

Additional file 1

Details of the assembly of the SSB system

Smart sustainable bottle (SSB) system for *E. coli* based recombinant protein production

Zhaopeng Li[1], Bettina Carstensen[1] and Ursula Rinas[1,2*]

1 Leibniz University of Hannover, Technical Chemistry – Life Science, Hannover, Germany

2 Helmholtz Centre for Infection Research, Braunschweig, Germany

*Corresponding author. Ursula.Rinas@helmholtz-hzi.de

Step 1 Assembly of cooling finger

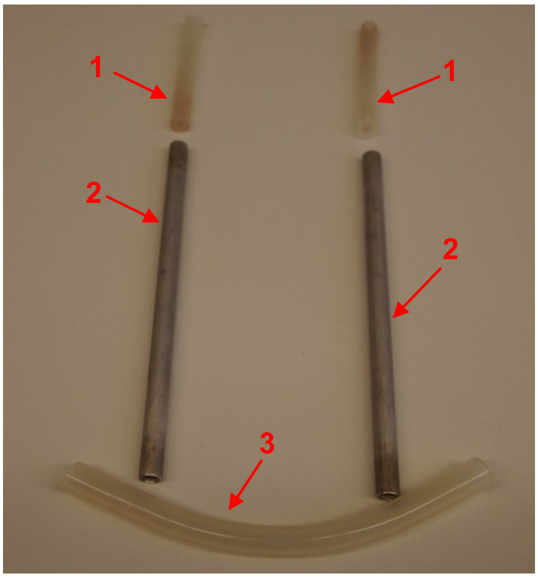

Figure 1

1: Silicone tube

Ø inner 5 mm, Ø outer 8 mm, wall thickness 1.5 mm, length 7 cm.

2: Stainless steel tube

Ø inner 6 mm, Ø outer 8 mm, wall thickness 1 mm, length 15 cm.

3: Silicone tube

Ø inner 6 mm, Ø outer 10 mm, wall thickness 2 mm, length 15 cm.

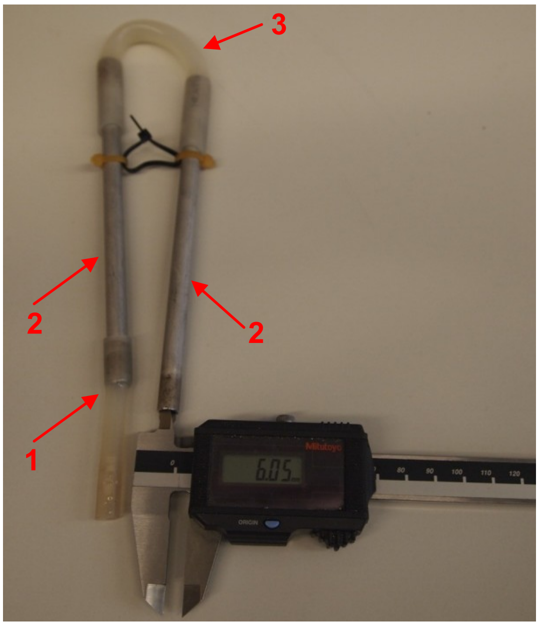

Figure 2

Link the silicone tubes (1), stainless steel tubes (2), and silicone tube (3). Fasten the stainless steel tubes by three cable ties to form a U shape as shown in Figures 2 and 3.

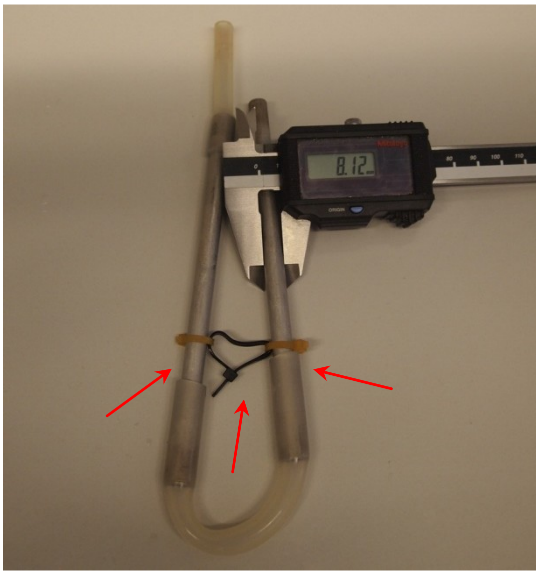

Figure 3

Step 1 Assembly of cooling finger

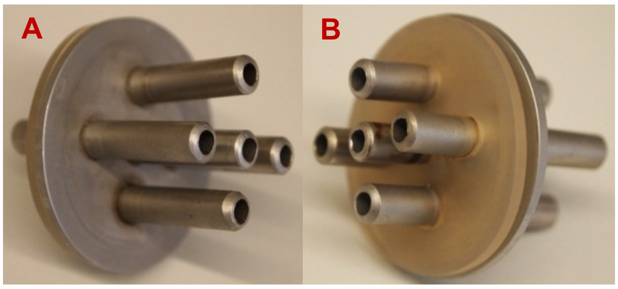

Figure 4

Self-made 5-port device (made by stainless steel) inner (lower) side without (A) and outer (upper) side with silicone gasket (B) is shown in Figure 4

Ø outer 41.5 mm (Figure 5A)

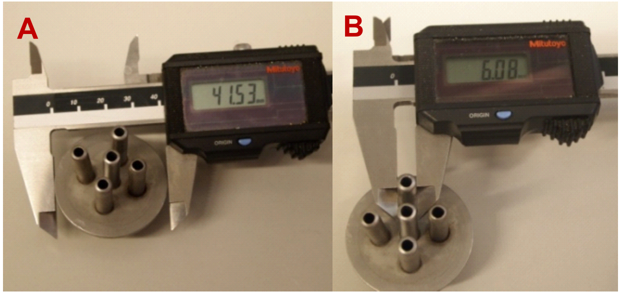

Figure 5

Stainless steel hose connections

Ø outer 6 mm (Figure 5B)

Ø inner 3.5 mm (Figure 6)

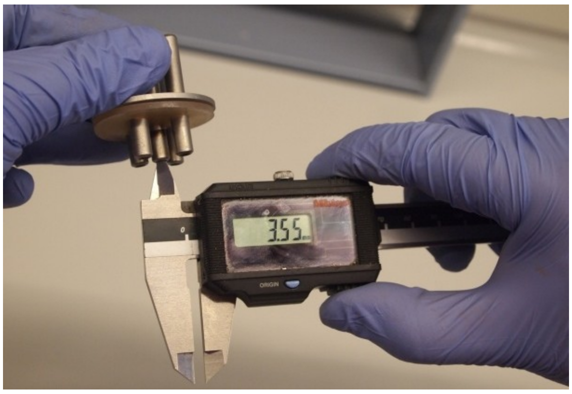

Figure 6

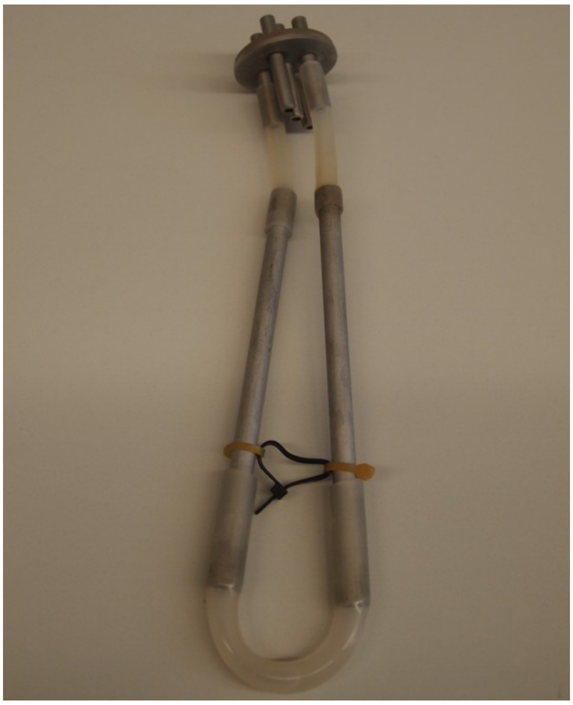

Figure 7

Connect the U shape cooling finger to the inner side of the 5-port device as shown in Figure 7

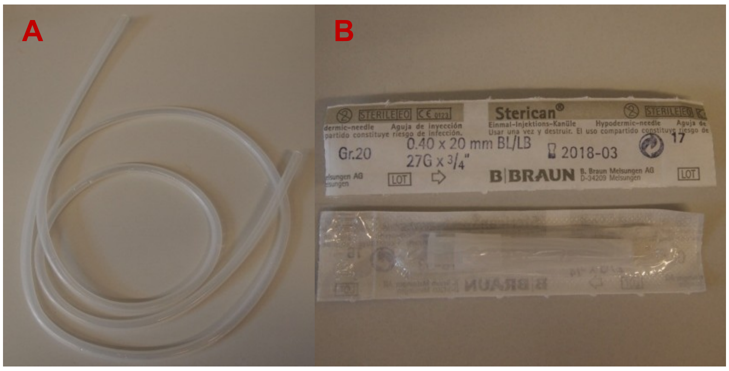

Figure 8

Step 2 Assembly of sparger

A: Silicone tube

Ø inner 4 mm, Ø outer 6 mm, wall thickness 1 mm, length 1.1 m.

B: Single-use needle

Ø outer 0.4 mm, length 20 mm.

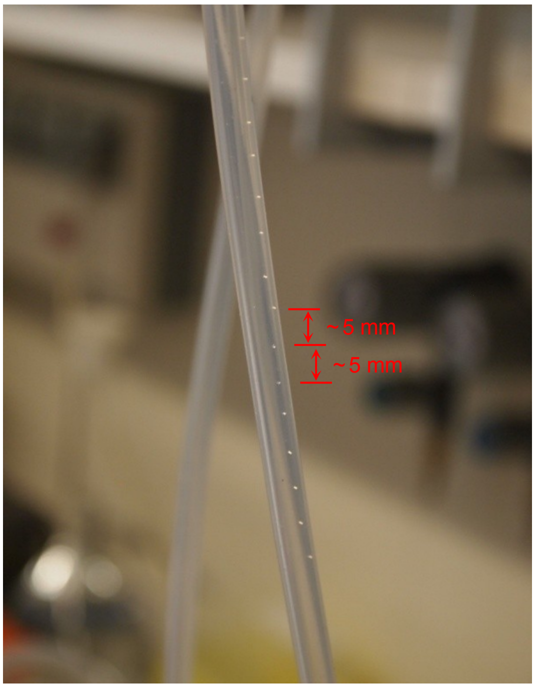

Figure 9

About every 5 mm on the silicone tube (as shown in Figure 9), four holes in cross shape (+) were made by two single-use needles as shown in Figure 10.

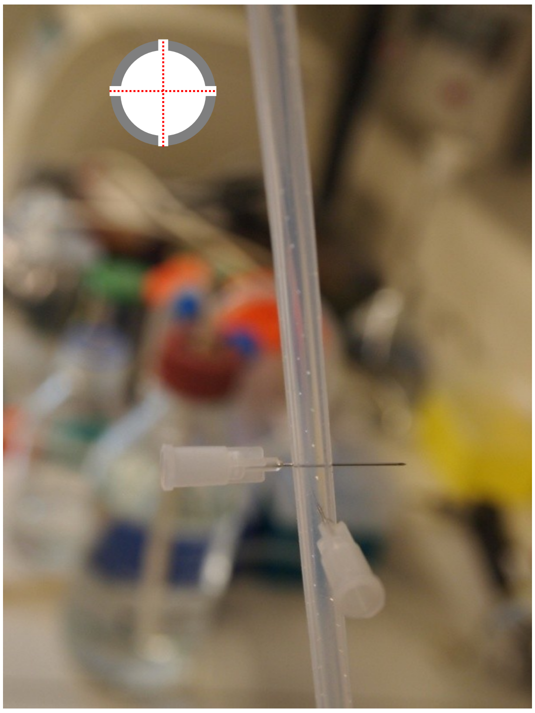

Figure 10

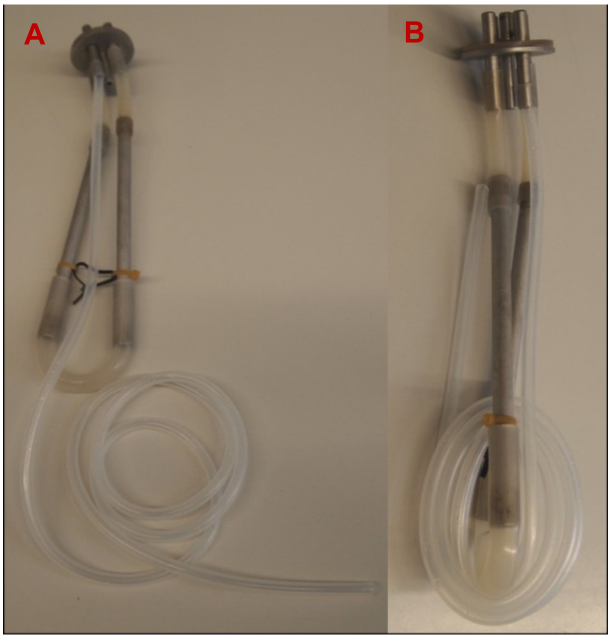

Figure 11

Step 2 Assembly of sparger

Connect the self-made sparger silicone tube to the inner side of the 5-port device (as shown in Figure 11A), which is already linked to the U shape cooling finger.

Wind the sparger silicone tube around the lower part of U shape cooling finger (as shown in Figure 11B).

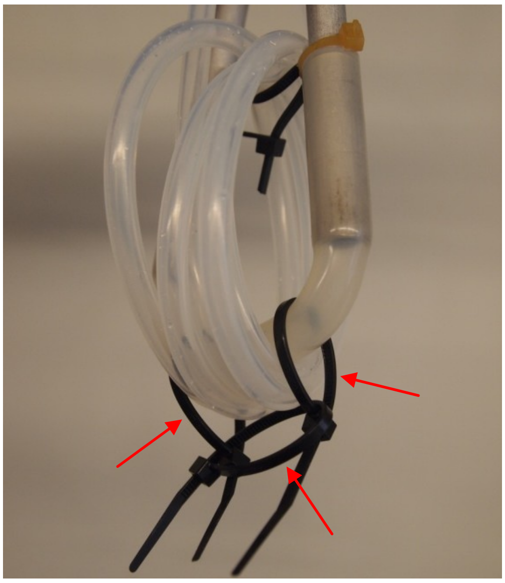
Figure 12

Fasten the sparger silicone tube by three cable ties to the U shape cooling finger as shown in Figure 12.

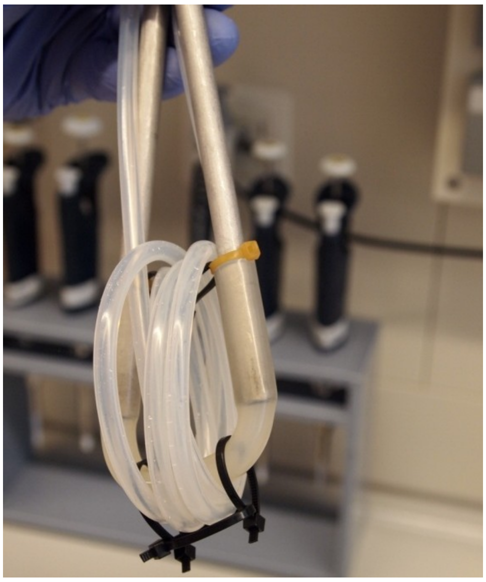
Figure 13

Cut off the unnecessary part of cable ties to finish the self-made sparger and cooling finger complex.

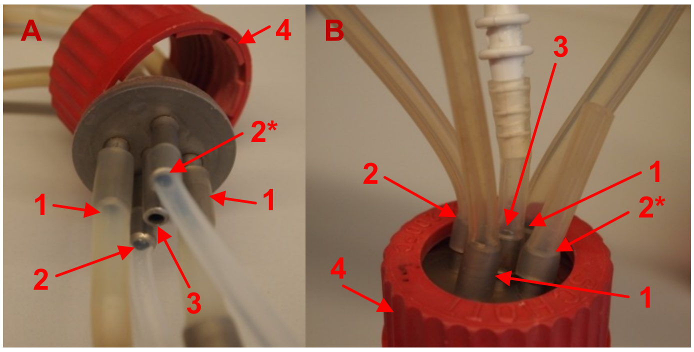

Figure 14

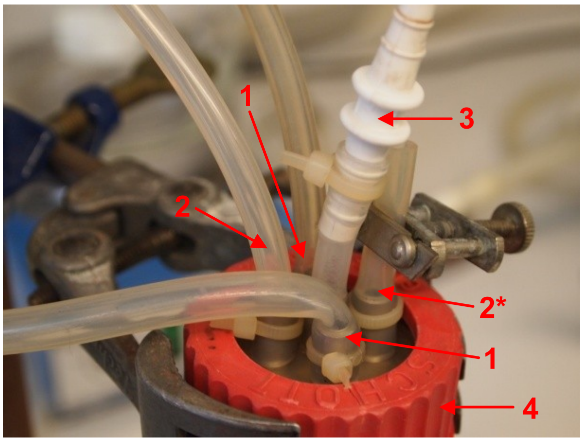

Figure 15

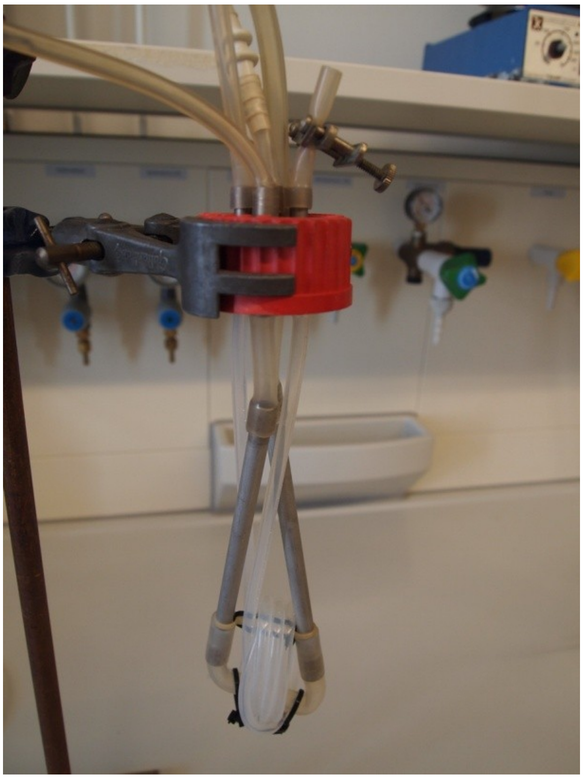

Figure 16

Step 3 Tubing connections

Connect the other end of sparger silicone tube (2*) to the inner side of 5-port device (as shown in Figure 14A)

Connect the outer side of 5-port device with silicone tube (as shown in Figure 14B)

Ø inner 4 mm, Ø outer 7 mm, wall thickness 1.5 mm

1 cooling finger

2 sparger silicone tube

3 off-gas

4 screw cap with aperture (GL 45)

Close the out-let (2*) of the sparger silicone tube to allow the air to go into the culture vessel through the holes made in Figure 10.

The sparger and cooling finger complex is ready to use.

Step 3 Assembly of the SSB system

A big magnetic stirrer bar (Ø 20 mm, length 50 mm, as shown in Figure 17) is used.

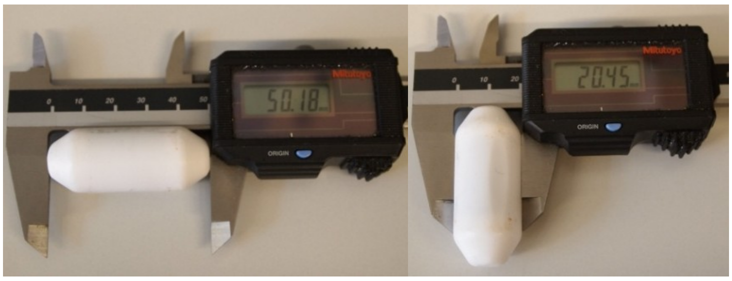

Figure 17

Put the magnetic stirrer bar in a 2 L Schott bottle. To facilitate sampling and addition of medium compounds, one sample port (**1**) and one liquid inlet port (**2**) can be added to the 2-L Schott bottle by a professional glassmaker (as shown in Figure 18).

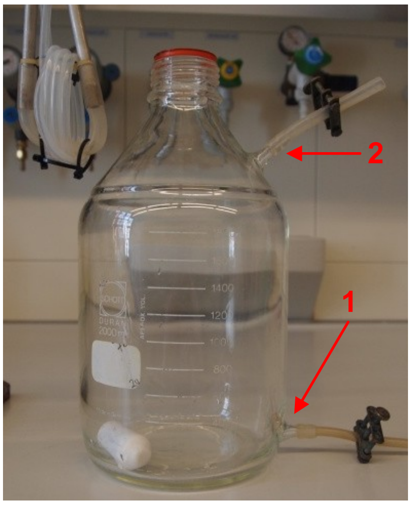

Figure 18

Put the sparger and cooling finger complex into the 2-L Schott bottle.

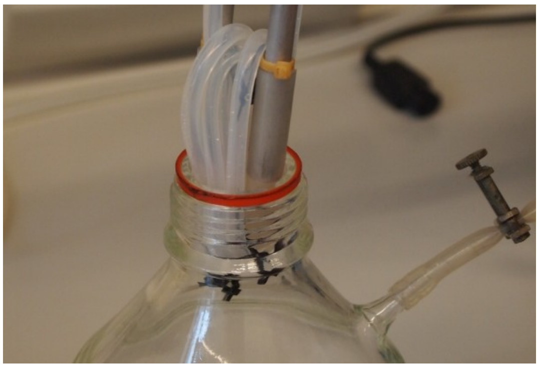

Figure 19

The SSB system is ready to use!!!!

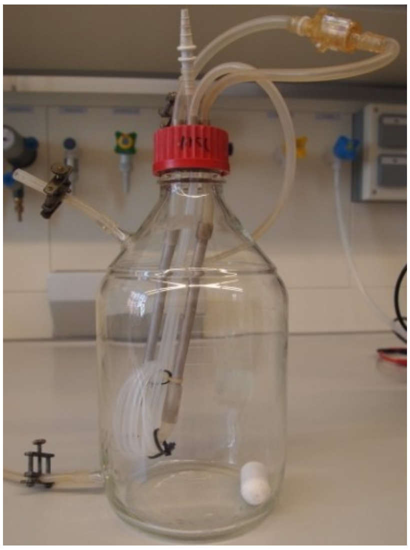

Figure 20

Step 4 Testing

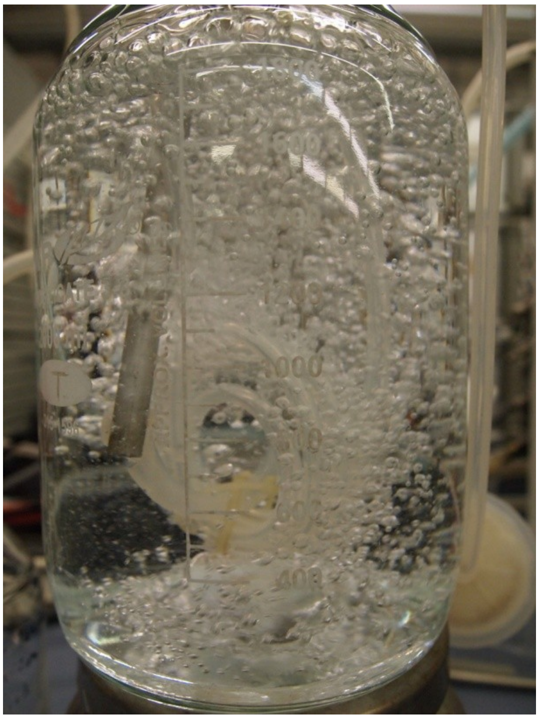

Figure 20

Before cultivation, testing of the SSB system for stirring stability, sparger performance, and potential leakage from the cooling finger is recommended.

The testing conditions are 1 L min^{-1} air, 500 rpm and water (as shown in Figure 20).

For the majority of cases, autoinduction cultivations using S-DAB (HNC) medium in the SSB system do not need sampling and the optional "booster" amino acids can be added in a clean bench. Thus, the sample port and the liquid inlet port (as shown in figure 18) are not mandatory. A normal 2 L Schott bottle (as shown in Figure 20) is sufficient.

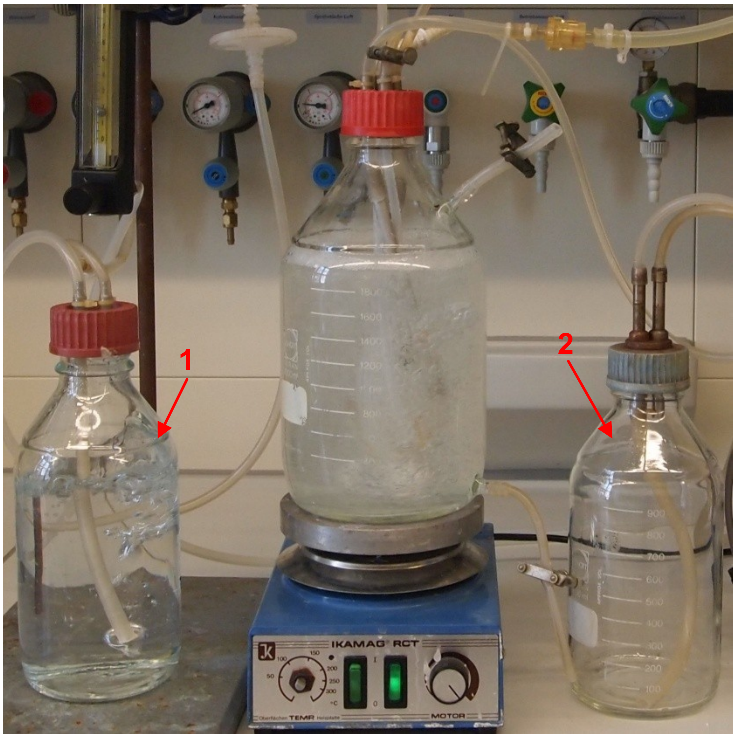

Figure 21

Due to the lack of an off-gas condenser liquid is lost by evaporation. To compensate the water loss, a bottle (**1**), which contains only water, is added to pre-wet the inlet air before going into the main culture bottle. As foaming can occur, even when antifoam is added, a safety bottle (**2**) should be added along the off-gas line.
