# Supplementary material for: Smart sustainable bottle (SSB) system for E. coli based recombinant protein production
Source: Microb Cell Fact. 2014 Nov 5;13:153. doi: 10.1186/s12934-014-0153-9 (PMC4226889; doi:10.1186/s12934-014-0153-9)
Supplement: Additional file 2: Figure S1 — hFGF-2 production in the SSB system and 30-L conventional bioreactor at 50% air saturation. Figures S2-S3. Adding “booster” amino acids to improve the soluble production of GST-GFP and TRX-hLIF in shake flask cultivation. Figures S4-S5. Adding “booster” amino acids to improve the soluble production of GST-GFP and TRX-hLIF in the SSB system. [file 12934_2014_153_MOESM2_ESM.pdf]

Additional file 2

Supplementary figures

Smart sustainable bottle (SSB) system for *E. coli* based recombinant protein production

Zhaopeng Li[1], Bettina Carstensen[1] and Ursula Rinas[1,2*]

1 Leibniz University of Hannover, Technical Chemistry – Life Science, Hannover, Germany

2 Helmholtz Centre for Infection Research, Braunschweig, Germany

*Corresponding author. Ursula.Rinas@helmholtz-hzi.de

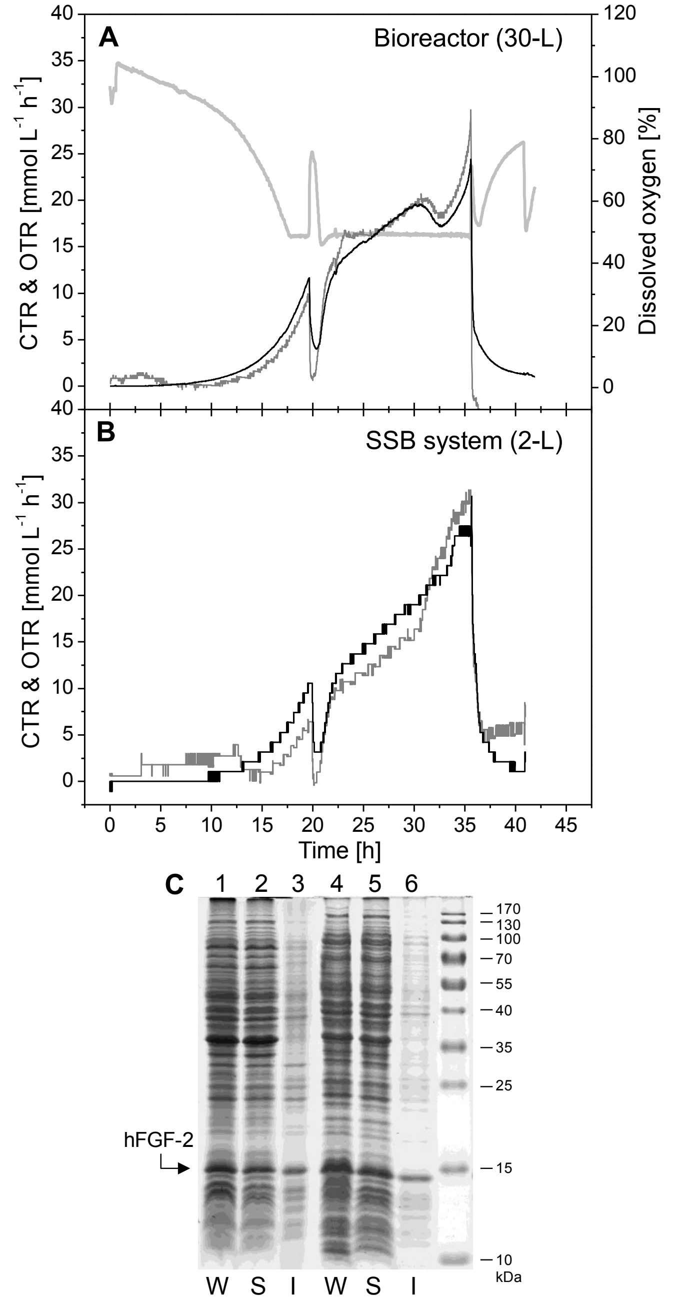

Figure S1 Production of hFGF-2 in a conventional 30-L bioreactor and the SSB system. Autoinduction cultivations were carried out in a 30-L bioreactor at controlled dissolved oxygen concentration (**A**) and the SSB system (**B**) for the production of hFGF-2. The carbon dioxide (CTR, black line) and oxygen transfer rates (OTR, gray line), and the dissolved oxygen concentration (light gray line) are shown. hFGF-2 production in bioreactor (lanes 1-3) and SSB system (lanes 4-6) were analyzed by SDS-PAGE (**C**). W: whole cell protein, S: soluble part, and I: insoluble part of whole cell protein.

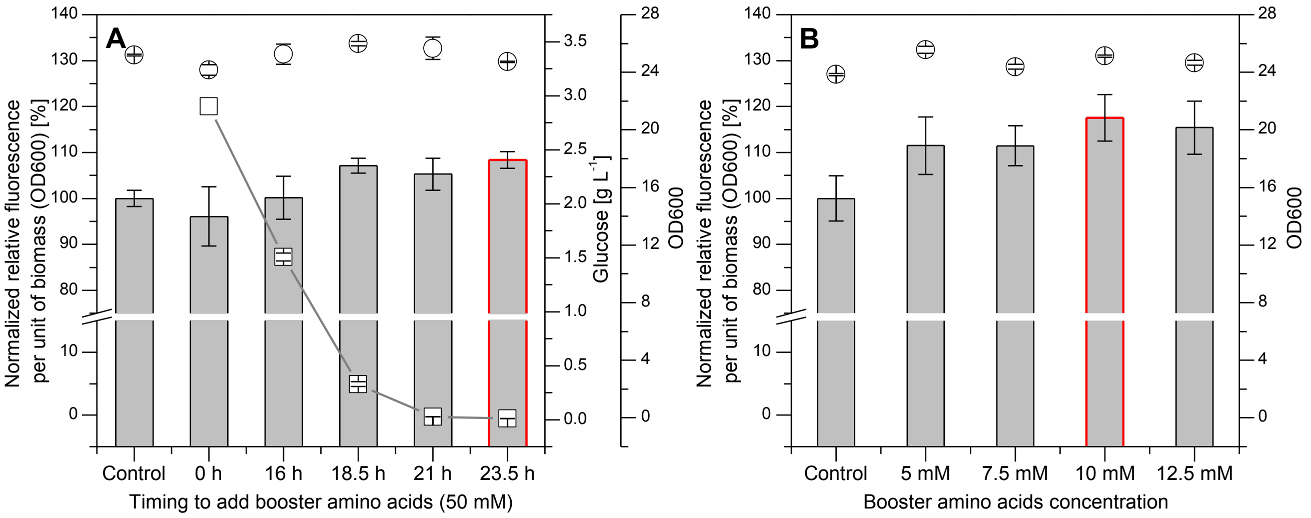

Figure S2 Adding "booster" amino acids to improve the soluble production of GST-GFP in shake flasks. "Booster" amino acids (L-arginine, L-asparagine, glycine, L-proline, L-leucine, and L-threonine; 5 mM each) were added to autoinduction cultivation (S-DAB (HNC) in shake flasks at 180 rpm and 23°C) at different time points (**A**). The relative fluorescence per unit of biomass (OD_{600}) normalized to the control run without "booster" amino acids addition (at 48 h, gray bar), the final cell densities (OD_{600} at 48 h, open circle), and glucose concentrations (open square) at the time point of "booster" amino acids addition are given (**A**). The optimal timing (0 g L^{-1} glucose) for "booster" amino acids addition is marked in red (**A**). When glucose concentrations reached zero, different concentrations of "booster" amino acids (5 to 12.5 mM for each amino acid) were added to autoinduction cultivation (S-DAB (HNC) in shake flasks at 180 rpm and 23°C) (**B**). The normalized relative fluorescence per unit of biomass (at 48 h, gray bar) and the final cell densities (OD_{600} at 48 h, open circle) are shown (**B**). The optimal concentration of "booster" amino acids (10 mM for each amino acid) is marked in red (**B**).

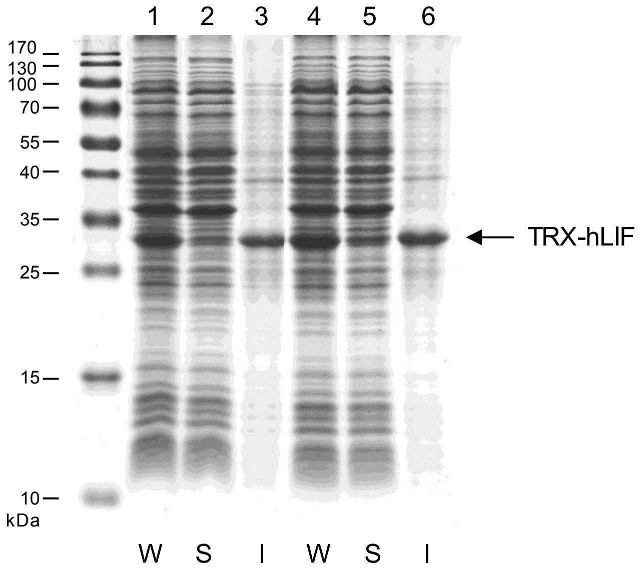

Figure S3 Adding "booster" amino acids to improve the soluble production of TRX-hLIF in shake flasks. "Booster" amino acids (L-arginine, L-asparagine, glycine, L-proline, L-leucine, and L-threonine; 10 mM each) were added to autoinduction cultivation (S-DAB (HNC) in shake flasks at 180 rpm and 23°C) when glucose concentrations reached zero. TRX-hLIF production in control run (without "booster" amino acids addition, final OD_{600}=18.6, lanes **1**-**3**) and cultivation with "booster" amino acids addition (final OD_{600}=20.4, lanes **4-5**) after 48 h cultivation were analyzed by SDS-PAGE. W: whole cell protein, S: soluble part, and I: insoluble part of whole cell protein. Cell pellets were disrupted by BugBuster™ Protein Extraction Reagent (Novagen, USA).

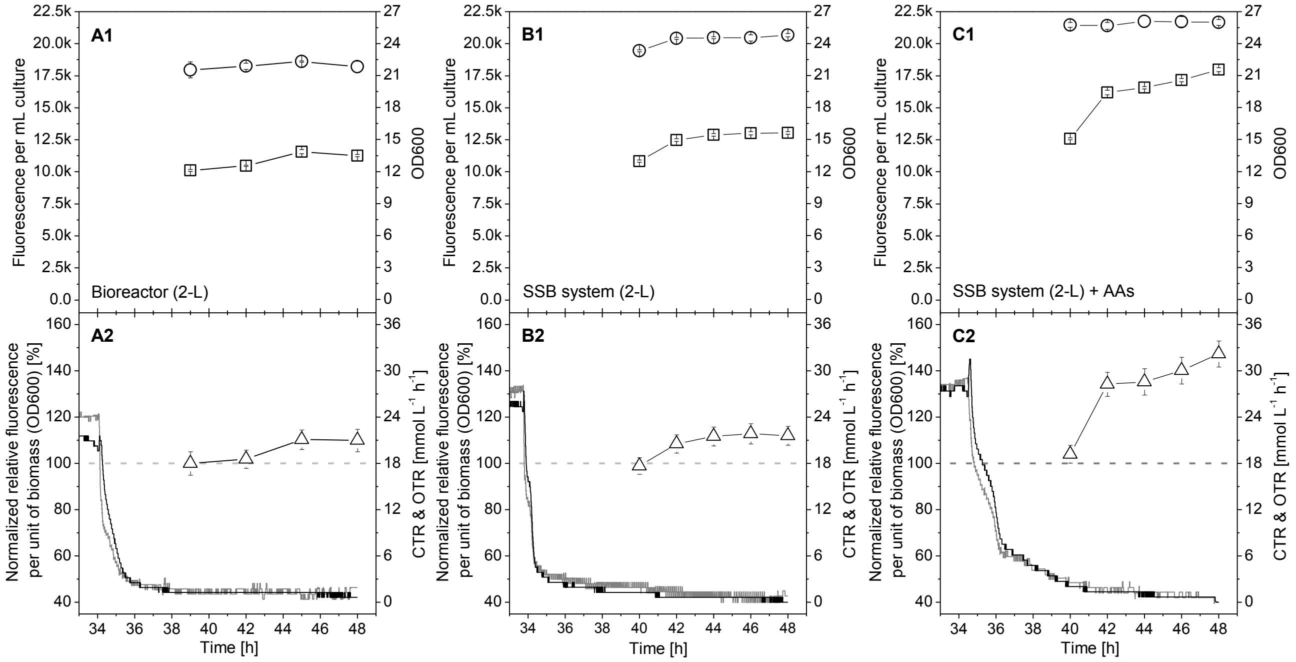

Figure S4 Adding "booster" amino acids to improve the soluble production of GST-GFP in the SSB system. Autoinduction cultivations using S-DAB (HNC) medium for the production of GST-GFP were carried out in 2-L bioreactor (**A1**-**A2**), SSB system (**B1**-**B2**), and SSB system with "booster" amino acids addition (**C1**-**C2**). The cell densities (OD_{600}, open circle) (**A1**-**C1**), the fluorescence per mL culture broth (open square) (**A1**-**C1**), the relative fluorescence per unit of biomass (OD_{600}) normalized to the value at 39 h in 2 L bioreactor as indicator of the specific amount of correctly folded GST-GFP (open triangle) (**A2**-**C2**), and the carbon dioxide (black line) and oxygen transfer rates (gray line) are shown (**A2**-**C2**). For the detailed cultivation profiles and SDS-PAGE analyses please refer to Figure 4.

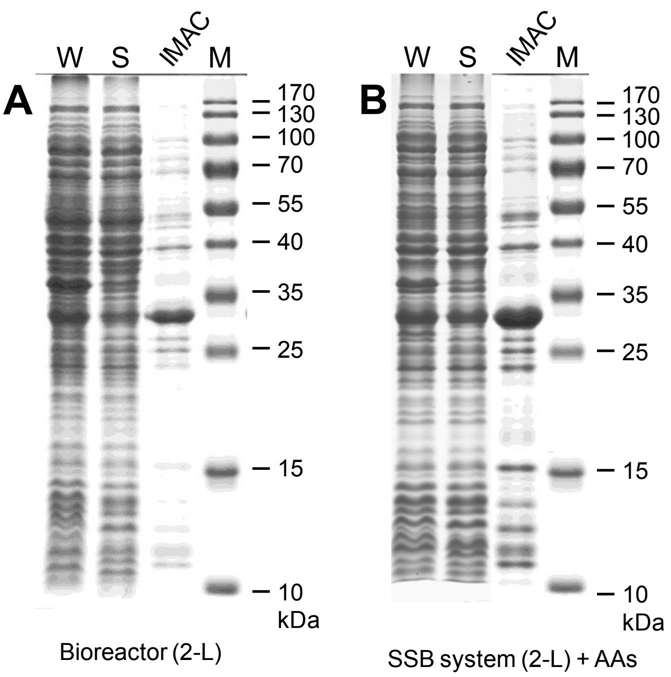

Figure S5 Adding "booster" amino acids to improve the soluble production of TRX-hLIF in the SSB system. Cells (indicated by **W**, whole cell) from cultivation in 2-L bioreactor (**A**) and with "booster" amino acids addition in SSB system (**B**) were disrupted by a high pressure homogenizer (M-110L; Microfluidics, USA). After centrifugation (20,000 g for 80 min), soluble cell lysate(s) (indicated by **S**) were loaded on HiTrap IMAC FF columns (GE Helthcare, UK) pre-charged with Zn^{2+}. After washing with 100 mM imidazole, TRX-hLIF was eluted using 250 mM imidazole (indicated by **IMAC**). For an unbiased comparison, handling of the biomass and purification was carried out under identical conditions, thus, cell pellet mass, volume, and concentration of cell suspension, volume of the IMAC TRX-hLIF eluate, and sample volumes for SDS-PAGE analysis were identical for samples from both cultivations. Chromatography was carried out as described previously [1]. For the detailed cultivation profiles please refer to Figure 4.

1. Tomala M, Lavrentieva A, Moretti P, Rinas U, Kasper C, Stahl F, Schambach A, Warlich E, Martin U, Cantz T, Scheper T: **Preparation of bioactive soluble human leukemia inhibitory factor from recombinant *Escherichia coli* using thioredoxin as fusion partner.** *Protein Expr Purif* 2010, 73:51-57.
