# Supplementary material for: Smart sustainable bottle (SSB) system for E. coli based recombinant protein production
Source: Microb Cell Fact. 2014 Nov 5;13:153. doi: 10.1186/s12934-014-0153-9 (PMC4226889; doi:10.1186/s12934-014-0153-9)
Supplement: Additional file 3: Table S1 — Literature survey of oxygen transfer capacities (kLa values) in shake flasks, single use culture vessels, and conventional bioreactors. [file 12934_2014_153_MOESM3_ESM.pdf]

Additional file 3

Supplementary table

Smart sustainable bottle (SSB) system for *E. coli* based recombinant protein production

Zhaopeng Li[1], Bettina Carstensen[1] and Ursula Rinas[1,2*]

1 Leibniz University of Hannover, Technical Chemistry – Life Science, Hannover, Germany

2 Helmholtz Centre for Infection Research, Braunschweig, Germany

*Corresponding author. Ursula.Rinas@helmholtz-hzi.de

Table S1 Literature survey of oxygen transfer capacities (k_La values) in shake flasks, single use culture vessels, and conventional bioreactors

Cultivation vessel	Working volume	Oxygenation condition	Baffled	k_La	Reference
250 mL Shake Flask	40 %	250 rpm (orbital shaking) in a shaker with amplitude of 1.6 cm	No	31 h^{-1}	[1]
250 mL Shake Flask	40 %	250 rpm (orbital shaking) in a shaker with amplitude of 1.6 cm	Yes	59 h^{-1}	[1]
10 L BIOSTAT® CultiBag RM system	10 %	Air flow was regulated between 0.1 and 0.6 vvm, 42 rpm rocking (10 °angle)	No	55 h^{-1}	[2]
10 L Wave Bioreactor® Cellbag	50 %	Air flow rate 0.1 vvm (with sparger), 40 rpm rocking (10 °angle)	No	60 h^{-1}	[3]
20 L Cylindrical Disposable Bioreactor	20 %	220 rpm (orbital shaking) in a shaker with amplitude of 5 cm	No	~ 80 h^{-1}	[4]
500 mL Shake Flask	10 %	160 rpm (orbital shaking) in a shaker with amplitude of 5 cm	No	127 h^{-1}	This work
500 mL Shake Flask	10 %	160 rpm (orbital shaking) in a shaker with amplitude of 5 cm	Yes	146 h^{-1}	This work
250 mL Shake Flask	20 %	300 rpm in a shaker. Shaking amplitude was not specified	Yes	150 h^{-1}	[5]
50 L BIOSTAT® CultiBag STR plus MO (Stirred Single-Use Bioreactor)	not specified	Oxygenation conditions for k_La measurement were not specified. Cultivation conditions are air flow rate of 5 to 20 L min^{-1}, agitation rate of 250 to370 rpm with a 6-blade disk impeller (bottom) and a 3-blade-segment impeller (top).	Yes	163 h^{-1}	[6]
2 L Bioreactor (glass)	100 %	Air flow rate 1 vvm, 500 rpm with two Rushton 6-blade impellers	No	162 h^{-1}	This work
**2 L SSB system**	**100 %**	**Air flow rate 1 vvm, 1100 rpm using a magnetic stirrer bar (Ø 20 mm, L 50 mm)**	**No**	**208 h^{-1}**	**This work**
10-L Stainless Steel Bioreactor	not specified	Oxygenation conditions for k_La measurement were not specified.	not specified	392 h^{-1}	[6]
2 L Bioreactor (glass)	100 %	Air flow rate 1 vvm, 1000 rpm with two Rushton 6-blade impellers	No	701 h^{-1}	This work
Stainless Steel Bioreactor	not specified	Superficial gas velocity (U_G) of 0.81 10^{-3} m/s, 300 to 800 rpm with a Rushton 6-blade impeller	Yes	~360 h^{-1} to ~1000 h^{-1}	[7]

References

1. Gupta A, Rao G: **A study of oxygen transfer in shake flasks using a non-invasive oxygen sensor.** *Biotechnology and Bioengineering* 2003, **84:**351-358.
2. Glazyrina J, Materne EM, Dreher T, Storm D, Junne S, Adams T, Greller G, Neubauer P: **High cell density cultivation and recombinant protein production with *Escherichia coli* in a rocking-motion-type bioreactor.** *Microb Cell Fact* 2010, **9:**42.
3. Mikola M, Seto J, Amanullah A: **Evaluation of a novel Wave Bioreactor(R) cellbag for aerobic yeast cultivation.** *Bioprocess and Biosystems Engineering* 2007, **30:**231-241.
4. Klockner W, Gacem R, Anderlei T, Raven N, Schillberg S, Lattermann C, Buchs J: **Correlation between mass transfer coefficient k_La and relevant operating parameters in cylindrical disposable shaken bioreactors on a bench-to-pilot scale.** *J Biol Eng* 2013, **7:**28.
5. Wittmann C, Kim HM, John G, Heinzle E: **Characterization and application of an optical sensor for quantification of dissolved O2 in shake-flasks.** *Biotechnol Lett* 2003, **25:**377-380.
6. Dreher T, Husemann U, Zahnow C, de Wilde D, Adams T, Greller G: **High cell density *Escherichia coli* cultivation in different single-use bioreactor systems.** *Chem Ing Tech* 2013, **85:**162-171.
7. Benadda B, Ismaili S, Otterbein M: **Relation of mechanical power to gas holdup and mass transfer in an agitated vessel.** *Chem Eng Technol* 1997, **20:**192-198.
