# Supplementary material for: Smart sustainable bottle (SSB) system for E. coli based recombinant protein production
Source: Microb Cell Fact. 2014 Nov 5;13:153. doi: 10.1186/s12934-014-0153-9 (PMC4226889; doi:10.1186/s12934-014-0153-9)
Supplement: Additional file 4 — Details of medium composition and preparation. [file 12934_2014_153_MOESM4_ESM.pdf]

Additional file 4

Details of medium composition and preparation

Smart sustainable bottle (SSB) system for *E. coli* based recombinant protein production

Zhaopeng Li[1], Bettina Carstensen[1] and Ursula Rinas[1,2*]

1 Leibniz University of Hannover, Technical Chemistry – Life Science, Hannover, Germany

2 Helmholtz Centre for Infection Research, Braunschweig, Germany

*Corresponding author. Ursula.Rinas@helmholtz-hzi.de

The composition of Luria-Bertani (**LB**) broth was as follows: 10 g L^{-1} tryptone, 5 g L^{-1} yeast extract, and 5 g L^{-1} NaCl. The pH was adjusted at pH 7 by NaOH before autoclaving. For solidification, 15 g L^{-1} agar was added.

The composition of Defined Non-inducing Broth (**DNB**) was as follows: 12 g L^{-1} glucose.H_2O, 1.2 g L^{-1} $MgSO_4.7H_2O$, 4 g L^{-1} $(NH_4)_2HPO_4$, 13.3 g L^{-1} KH_2PO_4, 1.7 g L^{-1} citric acid.H_2O, 100.8 mg L^{-1} Fe(III) citrate, 2.1 mg L^{-1} $Na_2MoO_4.2H_2O$, 1.4 mg L^{-1} $CoCl_2$, 12.3 mg L^{-1} $MnCl_2.2H_2O$, 1.2 mg L^{-1} $CuCl_2$, 3 mg L^{-1} H_3BO_3, 33.8 mg L^{-1} $Zn(CH_3COOH)_2.2H_2O$, 14.1 mg L^{-1} Titriplex III. The pH was adjusted at pH 6.8 by NaOH.

The composition of "Smart"-Defined Autoinduction Broth (High Nitrogen Content) (**S-DAB (HNC)**) was as follows: 3.23 g L^{-1} glucose.H_2O, 11.17 g L^{-1} glycerol, 8 g L^{-1} lactose.H_2O, 1.2 g L^{-1} $MgSO_4.7H_2O$, 4 g L^{-1} $(NH_4)_2HPO_4$, 13.3 g L^{-1} KH_2PO_4, 1.7 g L^{-1} citric acid.H_2O, 100.8 mg L^{-1} Fe(III) citrate, 2.1 mg L^{-1} $Na_2MoO_4.2H_2O$, 1.4 mg L^{-1} $CoCl_2$, 12.3 mg L^{-1} $MnCl_2.2H_2O$, 1.2 mg L^{-1} $CuCl_2$, 3 mg L^{-1} H_3BO_3, 33.8 mg L^{-1} $Zn(CH_3COOH)_2.2H_2O$, 14.1 mg L^{-1} Titriplex III. The pH was adjusted at pH 6.8 by ammonium hydroxide (~30%).

The composition of the DNB and S-DAB (HNC) media were described previously [1, 2]. To prevent precipitation during long-term storage, preparation methods were slightly modified as follows: the trace elements ($CoCl_2$, $MnCl_2.2H_2O$, $CuCl_2$, H_3BO_3, $Zn(CH_3COOH)_2.2H_2O$, and Titriplex III) were prepared as 2,000 times concentrated stock solution and stored at −20°C. $Na_2MoO_4.2H_2O$ was prepared as 5,000 times concentrated stock solution and stored separately at −20°C. Carbon source(s) (glucose, etc.), $MgSO_4$ and 2,000 times trace elements stock solution were prepared together as a combined stock solution (A group) which was heat-sterilized (121°C, 20 min). Ammonium and phosphate salts additionally including citric acid, ferric citrate, and $Na_2MoO_4.2H_2O$ were prepared as a combined stock solution (B group). After adjusting the pH to pH 6.8, the ammonium and phosphate salt stock solution (B group) can be heat-sterilized (121°C, 20 min).

References

1. Li Z, Kessler W, van den Heuvel J, Rinas U: **Simple defined autoinduction medium for high-level recombinant protein production using T7-based *Escherichia coli* expression systems.** *Appl Microbiol Biotechnol* 2011, 91:1203-1213.

2. Li Z, Nimtz M, Rinas U: **Optimized procedure to generate heavy isotope and selenomethionine-labeled proteins for structure determination using *Escherichia coli*-based expression systems.** *Appl Microbiol Biotechnol* 2011, 92:823-833

Table 1 2000 x trace element stock solution

Chemicals	mass
$CoCl_2$ (MW: 129.84)	0.7 g
$MnCl_2.2H_2O$ (MW: 161.87)	6.15 g
$CuCl_2$ (MW: 134.45)	0.6 g
H_3BO_3 (MW: 61.83)	1.5 g
$Zn(CH_3COO)_2.2H_2O$ (MW: 219.49)	16.9 g
Titriplex III (EDTA-Na_2, MW: 372.24)	7.05 g

Bring the final volume to 0.25 L with double distilled water (ddH_2O), aliquot the solution and store at −20°C.

Table 2 5000 x $Na_2MoO_4.H_2O$ stock solution

Chemicals	mass
$Na_2MoO_4.2H_2O$ (MW: 241.95)	1.05 g

Bring the final volume to 0.1 L with double distilled water (ddH_2O), aliquot the solution and store at −20°C.

Table 3 DNB medium A group (0.5 L)

Chemicals and stock solution	mass & volume
glucose.H_2O (MW: 198.17)	60 g
$MgSO_4.7H_2O$ (MW: 246.47)	6 g
2000 x trace element stock solution	2.5 mL

Bring the final volume to 0.5 L with double distilled water (ddH_2O) and autoclave at 121°C. Long term storage at room temperature is possible. The color of the solution may change without having negative effects.

Table 4 DNB medium B group (5 L)

Chemicals and stock solution	mass & volume
$(NH_4)_2HPO_4$ (MW: 132.06)	22.22 g
KH_2PO_4 (MW: 136.09)	73.89 g
citric acid.H_2O (MW: 210.1)	9.44 g
Fe(III) citrate (MW: 244.94)	0.56 g
NaOH (MW: 40)	15 g
5000 x $Na_2MoO_4.H_2O$ stock solution	1.11 mL

Add 4 L double distilled water (ddH_2O), use a hot plate magnetic stirrer for heating (about 60 to 80°C), and mix overnight to dissolve the Fe(III) citrate. After cooling to room temperature, adjust pH at pH 6.8. Bring the final volume to 5 L and autoclave at 121°C. Long time storage at room temperature is possible.

For preparation of 100 mL DNB medium, mix 10 mL A group, 90 mL B group and appropriate antibiotic in a clean bench.

Table 4 S-DAB medium **A group** (5 L)

Chemicals and stock solution	mass & volume
glucose.H_2O (MW: 198.17)	53.83 g
glycerol (MW: 92.09)	186.10 g
lactose.H_2O (MW: 360.31)	133.33 g
$MgSO_4.7H_2O$ (MW: 246.47)	20.00 g
2000 x trace element stock solution	8.33 mL

Bring the final volume to 5 L with double distilled water (ddH_2O) and autoclave at 121°C. Long time storage at room temperature is possible. The color of the solution may change without having negative effects.

Table 5 S-DAB (HNC) medium B group (10 L)

Chemicals and stock solution	weight & volume
$(NH_4)_2HPO_4$ (MW: 132.06)	57.14 g
KH_2PO_4 (MW: 136.09)	190 g
citric acid.H_2O (MW: 210.1)	24.29 g
Fe(III) citrate (MW: 244.94)	1.44 g
5000 x $Na_2MoO_4.H_2O$ stock solution	2.86 mL

Add 8 L double distilled water (ddH_2O), use a hot plate magnetic stirrer for heating (about 60 to 80°C), and mix overnight to dissolve the Fe(III) citrate. After cooling to room temperature, adjust pH at pH 6.8 with appr. 55 ml ~30% ammonium hydroxide solution. Bring the final volume to 10 L and autoclave at 121°C. Long term storage at room temperature is possible.

For preparation of 1 L S-DAB (HNC) medium, mix 0.3 L A group, 0.7 L B group and appropriate antibiotic in a clean bench. For cultivation in SSB system or bioreactor, e.g. 2 L working volume, pour 1.4 L B group and 0.5 mL antifoam in SSB or bioreactor, prepare the cultivation vessel (e.g. calibration of pH and dissolved oxygen probes for the bioreactor) and autoclave at 121°C (previously prepared sterilized B group stock solution can be re-autoclaved). Mix appropriate antibiotic with 0.6 L A group in a sterilized container in a clean bench (re-autoclaving A group is not recommended). Aseptically transfer this mixture to the sterilized SSB system or bioreactor.

Table 6 "Booster" amino acids stock solution (125 mM for each amino acid, 1 L)

Chemicals	weight
L-arginine (MW: 174.2)	21.78 g
L-asparagine (MW: 132.12)	16.52 g
glycine (MW: 75.07)	9.38 g
L-proline (MW: 115.13)	14.39 g
L-leucine (MW: 131.17)	16.40 g
L-threonine (MW: 119.12)	14.89 g

Add 0.8 L double distilled water (ddH_2O) and use a magnetic stirrer to mix overnight. Then adjust pH at pH 7.5 and bring the final volume to 1 L. Sterilize by filtration (0.2 µm) and store at 4°C.
